# Influence of Graphene Nanoplates on Dispersion, Hydration Behavior of Sulfoaluminate Cement Composites

**DOI:** 10.3390/ma15155357

**Published:** 2022-08-03

**Authors:** Kai Cui, Jun Chang, Mohanad Muayad Sabri Sabri, Jiandong Huang

**Affiliations:** 1 School of Civil Engineering, Dalian University of Technology, Dalian 116024, China; jiancaick@mail.dlut.edu.cn; 2 Peter the Great St. Petersburg Polytechnic University, 195251 St. Petersburg, Russia; mohanad.m.sabri@gmail.com; 3 School of Mines, China University of Mining and Technology, Xuzhou 221116, China

**Keywords:** sulfoaluminate cement, graphene nanoplates, dispersion, hydration, hydration kinetics

## Abstract

Sulfoaluminate cement (SAC) is a low carbon ecological cement with good durability and is widely used in various projects. In addition, graphene nanoplates (GNPs) have excellent thermal, electrical, and mechanical properties and are excellent nano-filler. However, the hydration behavior of GNPs on SAC is still unclear. In this paper, the effect of GNPs on SAC hydration was investigated by isothermal calorimetry, and the hydration kinetic model and hydration kinetic equation of SAC was established, explaining the differences in cement hydration processes with and without GNPs on SAC based on a hydration kinetic model. Results indicate that the hydration exotherm of SAC mainly includes five stages: the initial stage, the induction stage, the acceleration stage, the deceleration stage, and the stable stage. The addition of GNPs promoted the hydration exotherm of SAC and accelerated the hydration reaction. Different from the hydration reaction of Portland cement, the hydration reaction of SAC is mainly a diffusion–reaction process.

## 1. Introduction

The greenhouse effect is increasing daily with the rapid development of the global economy and industry. The emission of CO_2_ is the leading cause of the greenhouse effect. The emission of CO_2_ has caused a massive threat to the global ecological environment, such as global warming, melting glaciers, sea level rise, species extinction, and extreme weather [1]. To reduce CO_2_ emissions, the international community proposes to achieve carbon neutrality in 2045 and 2050, respectively. China proposes to achieve carbon peaking in 2030 and carbon neutrality in 2060. It is imminent to reduce CO_2_ emissions and utilize CO_2_ [2,3]. The world produces more than 10 billion tons of CO_2_ every year, of which the CO_2_ emission of cement accounts for 5–8% [4]. In recent years, China’s total CO_2_ emissions have exceeded 11 billion tons, of which the CO_2_ emissions from cement and steel account for more than 50%. In 2021, China’s annual cement production reached 2.38 billion tons, and every ton of ordinary Portland cement (OPC) released 1 ton of CO_2_.

The cement industry is facing enormous pressure to reduce carbon emissions. Ecological low carbon cement has become the main direction of current cement development. Ecological refers to saving natural resources and being friendly to the environment; low carbon refers to reducing CO_2_ emissions. Currently, the reduction in CO_2_ emissions in cement production mainly includes four aspects: one is to use low calcium clinker minerals, the other is to reduce the use of carbonate raw materials, and the third is to use low-heat firing. Compared with OPC, sulfoaluminate cement has high early strength, fast setting and hardening, and excellent durability [5,6]. In the raw meal, using less limestone is in line with the use of low calcium clinker minerals, and the clinker firing temperature is 150–200 °C lower than OPC. As an ecological low carbon cement, SAC is widely used in emergency and construction, repair and reinforcement, marine engineering, military engineering, and unique engineering [7,8]. The fourth aspect is the recycling and reuse of waste and taking the road to sustainable development, such as recycled aggregate concrete, geopolymers, and various wastes, improving concrete strength and predicting concrete’s strength development [9]. Saleh et al. studied two kinds of wastes, cement kiln dust (CKD) and poly(styrene) (PS), which were added to Portland cement as additives to prepare lightweight bricks. It is a produced low carbon eco-economic lightweight bricks [10]. Saleh et al. studied polyvinyl chloride (PVC) pipe debris and asphalt as cement additives to improve cement-based materials’ mechanical properties and radiation shielding efficiency. The results show that cement composites containing PVC or asphaltenes have the best radiation shielding properties for stabilizing radioactive waste [11].

Cement-based materials are the most widely used building materials [12,13]. With the development of the construction and civil engineering industries, higher requirements have been placed on cement-based materials’ mechanical properties and durability. The high brittleness and high porosity of cement-based materials severely limit their use of cement-based materials. Therefore, many researchers have completed a lot of research on strengthening and toughening cement-based materials, such as adding nanomaterials such as carbon tubes [14,15,16], limestone powder [17], graphene [18], whiskers [19], and steel fibers [20]. As a two-dimensional carbon material, graphene nanoplates (GNPs) have a thickness of less than 100 nm and a diameter of several microns [21]. It has excellent mechanical properties, electrical properties, thermal properties, and an enormous specific surface area [22,23,24]. It is considered an ideal additive filler powder for improving cement-based materials. Deng et al. studied the effect of GNPs on the hydration of OPC. Due to the early sufficient ion exchange, GNPs accelerated the hydration reaction and generated more hydration products [25]. Dalal et al. found that GNPs can significantly improve cement-based materials’ flexural strength, tensile strength, and compressive strength [26]. Zhao et al. found that adding GNPs can affect the elastic modulus of cement-based materials, effectively suppressing the autogenous shrinkage and drying shrinkage of cement-based materials [27]. Tang et al. studied the effect of GNPs on the hydration of C_3_S and found that GNPs could adsorb C_3_S, prolong the dissolution and induction periods of C_3_S, and delay hydration [28]. Bhojaraju et al. studied the effect of different carbon-based materials, including graphene and graphene oxide, on the hydration kinetics of slag-modified cement and found that the improvement in compressive strength of cement-based materials stems from the increase in the degree of hydration [29]. Snellings et al. studied the hydration kinetics of ternary cement composed of clinker, slag, and limestone with different water–cement ratios. The results showed that the water–cement ratio affects the later hydration degree of slow-reacting phases such as belite [30]. In addition, some researchers use the Krstulovic–Dabic kinetic model to study the hydration process of cement. Zhou et al. used the Krstulovic–Dabic kinetic model to study the kinetics of the mixed cement containing bauxite tailings. The study showed that the kinetic model could effectively simulate the hydration process of the composite cement containing bauxite tailings [31]. Han et al. characterized the hydration processes of cement-based materials based on the kinetic hydration model. The results showed that the hydration processes were NG, I, and D stages, respectively [32]. Meng et al. studied the effect of NS on the heat of hydration of cement mortar, and the study showed that NS could shorten the phase I stage of the phase boundary reaction and reduce the D stage of the diffusion–reaction [33]. Zhang et al. simulated the hydration process of the cement–quicklime system and found that when the CaO content is low, it conforms to the Krstulovic–Dabic kinetic model. The model is not applicable when the CaO content exceeds 30% [34].

We found from previous studies that GNPs improve the mechanical properties of cement-based materials to varying degrees, including compressive strength, flexural strength, and elastic modulus. The research conclusions on the effect of GNPs on the hydration of cement-based materials are contradictory. Based on the Krstulovic–Dabic kinetic model, there is no unified standard for the study of cement hydration, and there are different conclusions based on various research factors. Research factors limit the applicability of the kinetic model, and the three hydration processes of the model do not apply to all cement hydrations. There is currently no research on the effect of GNPs on the hydration of SAC. It is vital to study the hydration of SAC based on the Krstulovic–Dabic kinetic model, which is beneficial for obtaining SAC’s dynamic hydration mechanism. This paper firstly explores the influence of dispersants on the dispersion of GNPs, and then focuses on the impact of GNPs in the hydration of sulfoaluminate cement-based materials. The hydration exothermic process of SAC and the effect of GNPs on the hydration exothermic process of SAC were studied, and the hydration kinetic model and hydration kinetic equation of SAC was established. We can explain the differences in cement hydration processes with and without GNPs on SAC based on a kinetic hydration model.

## 2. Materials and Methods

### 2.1. Materials

Sulfoaluminate cement is produced by Tangshan Polar Bear Building Materials Co., Ltd., Tangshan, China. The physical properties and chemical composition are shown in Table 1 and Table 2. Graphene was produced by XG Science, Inc. (Lansing, MI, USA), and the physical parameters are shown in Table 3. Polycarboxylate superplasticizer provided by Chongqing Sansheng Building Materials Co., Ltd. (Chongqing, China), with a water-reducing efficiency of 30%. Surfactant is used to disperse graphene. The surfactant includes SDBS (Sodium dodecylbenzene sulfonate), PVP (polyvinylpyrrolidone), and CTAB (cetyltrimethylammonium bromide); the defoamer is TBP (tributyl phosphate), provided by Tianjin Chemical Reagent Factory (Tianjin, China). In this experiment, the dosage of GNPs is 0, 0.03%, 0.06%, and 0.09% of the cement weight; the sample names are marked as G1, G2, G3, G4, and the water to cement ratio is 0.4.

### 2.2. The Preparation Process and Test Parameters

The dispersion of GNPs adopts a combination of non-covalent surface modification, mechanical stirring, and ultrasonic dispersion. First, pour the weighed surfactant into 50 g of distilled water, stir with a glass rod until the surfactant is completely dissolved, then pour the weighed 5 mg of GNPs into the solution, stir with a glass rod for 1 min, and then sonicated 10 min, the output power is 360 W, the dispersibility of GNPs is characterized by ultraviolet spectrophotometer, and the amount of surfactant in the optimal dispersion state of graphene is determined. According to the standard JC/T 729-2005, mix the best dispersed GNPs suspension, superplasticizer, defoamer, and cement, stir at low speed and high speed for 3 min, respectively, and finally drop the mixed paste into an ampoule bottle to test the heat of hydration. The remaining paste was poured into a 4 × 4 × 16 cm^3^ mold. After vibrating and compacting, the standard curing was performed for 1 d, and the mold was removed and cured for 28 days. UV spectrophotometer (T6, PERSEE Instruments, America) characterizes the dispersion effect of GNPs; Isothermal calorimetry (Tam air C80, TA Instruments, America) monitors the heat flow and total heat release for 24 h.

## 3. Results and Analysis

### 3.1. Dispersion of GNPs

UV–VIS with a wavelength of 260 nm was used to determine the optimal dispersant dosage; based on Lambert–Beer law, the dispersion degree of GNPs in the solution was characterized. The absorbance A was calculated by Equation (1) [35].

(1) $$A=\log(\frac{I_{r}}{I_{s}})=ECL$$

where *E* is a constant, *C* is solution concentration, and *L* is the optical path length. It can be seen that when the optical path length *L* is constant, the absorbance *A* is proportional to the solution concentration *C*.

**Figure 1:**
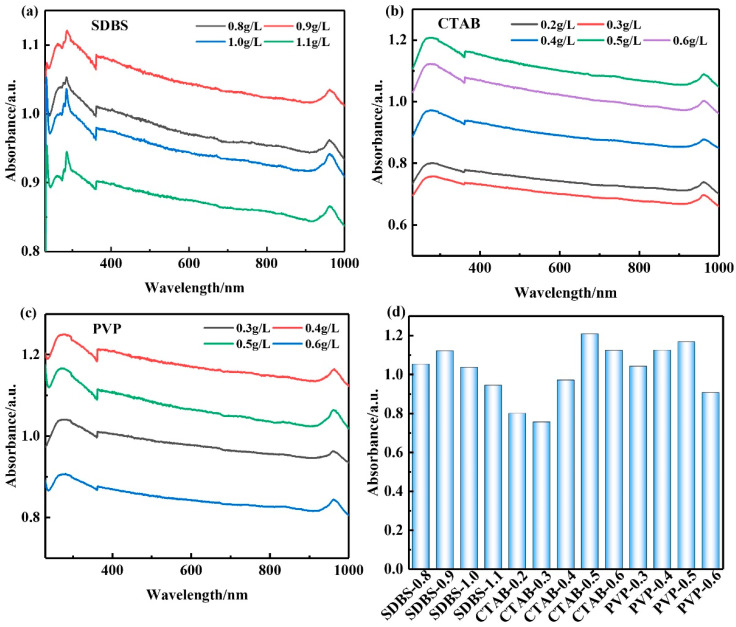
UV–VIS spectra of GNPs with different surfactant concentrations; (**a**) SDBS, (**b**) CTAB, (**c**) PVP, (**d**) maximum absorbance.

**Figure 2:**
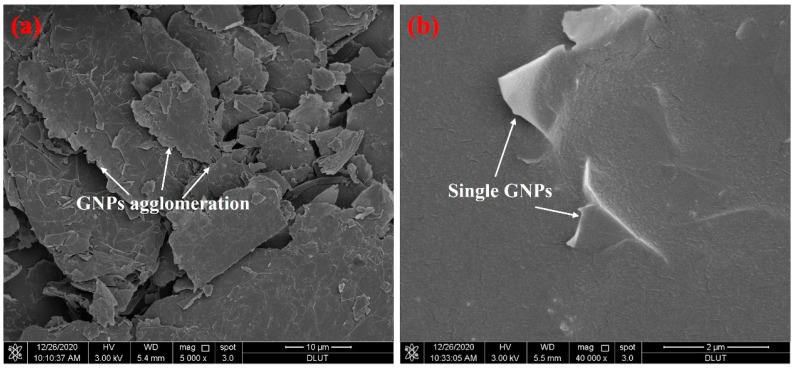
SEM of GNPs; (**a**) before dispersion, (**b**) after dispersion.

Figure 1 shows the UV–VIS spectra of GNPs mixed with different concentrations of different surfactants. Surfactants can improve the dispersion performance of GNPs in aqueous solutions to various degrees. The absorbance of GNPs increased and then decreased along with the increase in surfactant concentration, and there was a maximum absorbance. It can be seen from Figure 1d that when the concentration of SDBS is 0.9 g/L, the concentration of CTAB is 0.5 g/L, and the concentration of PVP is 0.4 g/L, which corresponds to the absorbance reaching maximum, the dispersion effect is better, among which CTAB has the largest absorbance. We use a CTAB concentration of 0.5 g/L to disperse GNPs for subsequent experiments. Figure 2 shows the SEM of graphene before and after dispersion. GNPs are bent and agglomerated together and form a block before dispersion; after surfactant, mechanical stirring, and ultrasonic dispersion, the block agglomeration of GNPs is dispersed; the thickness of graphene is reduced, the size is reduced, and there is no accumulation of large chunks.

### 3.2. Hydration Behavior

#### 3.2.1. Heat of Hydration

**Figure 3:**
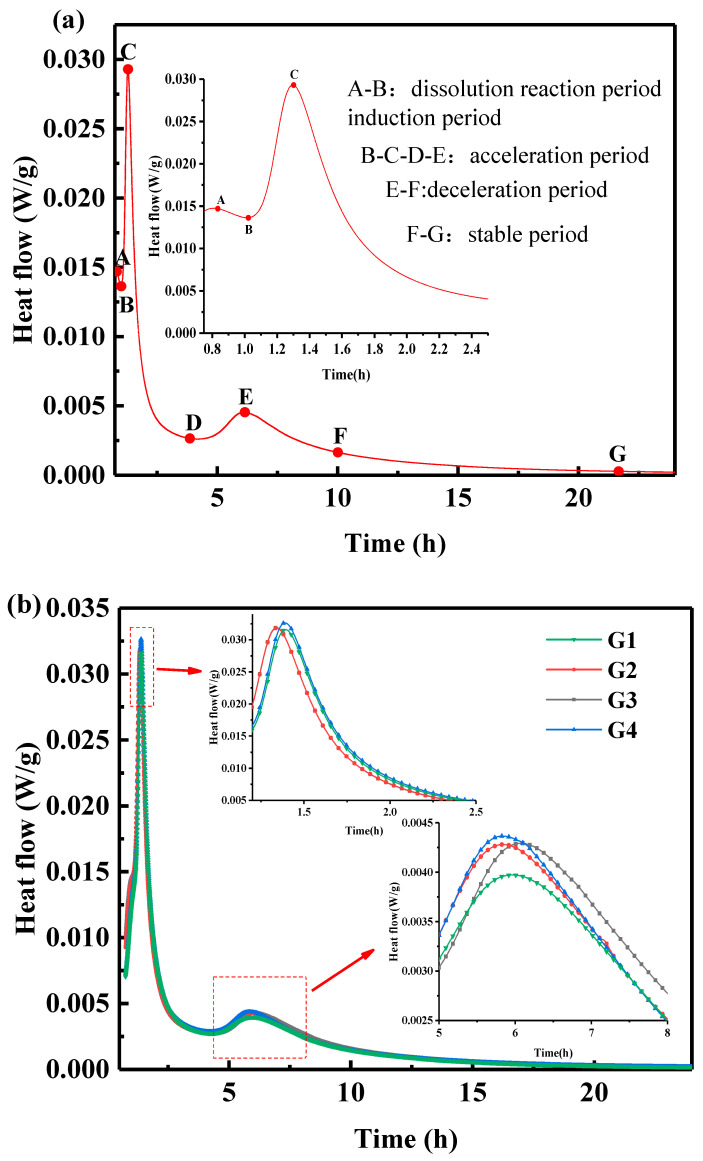

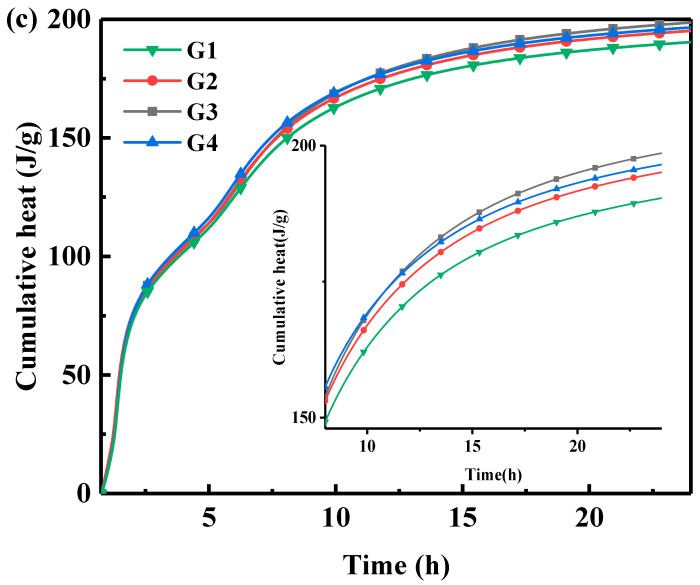
Hydration heat curves of sulfoaluminate cement-based materials with and without GNPs, (**a**) hydration stage, (**b**) hydration heat, and (**c**) cumulative heat.

The heat of hydration is an important physical parameter for evaluating the hydration of cement-based materials [5]. In order to explore the effect of GNPs on the hydration process of SAC, the heat of hydration of SAC with different dosages of GNPs was tested, as shown in Figure 3. According to the characteristics of hydration exothermic rate change of SAC, the hydration exothermic process can be divided into five reaction stages, as shown in Figure 3a [5]. The AB stage is the initial period and the induction period. After the cement particles are in contact with water, a small amount of heat is released. At this time, a small amount of AFt is generated. The hydration rate decreases, and the cement particles enter the induction period. The induction period is short, the hydration enters the acceleration period, and the B-C-D-E stage is the acceleration period. Two exothermic hydration peaks can be observed in the acceleration period, which can be divided into the initial and final stages of acceleration. In the initial stage of acceleration, due to the rupture of the coating layer during the induction period, C_4_A_3_Š rapidly contacted the solution and generated more AFt. The first exothermic peak corresponds to the formation of AFt and the consumption of C_4_A_3_Š, hydration entered the late acceleration stage, and a second exothermic peak appeared. Hydration products increased, filling unreacted particles and paste along with the growth of crystals, the paste lost plasticity to a certain extent and the hydration into a deceleration period.

The EF stage is the deceleration stage. Hydration products formed a coating layer along with the hydration, the porosity decreased, the resistance to the passage of water molecules increased, and the hydration heat release rate decreased. Then, the hydration enters a stable stage; at this stage, the exothermic rate of hydration is slow, the porosity of the paste is further reduced, and water molecules can hardly pass through the hydration products. Figure 3b is the curve of the hydration exothermic rate of samples with and without GNPs. The curves have two exothermic peaks, and the first exothermic peak of G1, G2, G3, and G4 appeared at 1.394 h, 1.344 h, 1.339 h, and 1.389 h, respectively. Adding GNPs promoted the hydration of the matrix, attributed to the nucleation effect of GNPs, and similar conclusions were obtained in other studies [23]. The second exothermic peaks of G1, G2, G3, and G4 appeared at 5.874 h, 5.783 h, 6.003 h, and 5.755 h, respectively; Figure 3c shows each group of samples’ cumulative hydration heat release for 24 h, the cumulative heat of each group is 189.3 J/g, 195.1 J/g, 199.7 J/g, and 196.5 J/g, respectively. 

#### 3.2.2. Hydration Kinetics

According to the hydration characteristics of cement-based materials, Kondo proposed a kinetic hydration formula, as shown in Equations (2) and (3).

(2) $${\left[1-{\left(1-\alpha \right)}^{\frac{1}{3}}\right]}^{N}=K(t-t_{0})$$

(3) $$In{\left[1-{\left(1-\alpha \right)}^{\frac{1}{3}}\right]}^{}=\frac{1}{N}InK+\frac{1}{N}In(t-t_{0})$$

where *α* is the degree of hydration, *N* is a constant related to the hydration mechanism, and the values differ at different stages. *K* is the hydration rate constant, and *t*_0_ is the end time of the induction period. When *N* is greater than or equal to 2, the hydration reaction depends on the diffusion rate of water molecules through the product layer, which is controlled by the diffusion–reaction. When *N* is 1, the hydration reaction depends on the solid–liquid phase reaction, which is determined by the phase boundary reaction control; when *N* is less than 1, the hydration reaction is mainly controlled by crystal nucleation growth.

The Kstulovic–Dabic model divides each stage in detail. The Kstulovic–Dabic model reflects the relationship between the degree of hydration and the hydration heat release time, and the kinetic hydration equation mainly includes three stages [36].

Crystal nucleation and crystal growth (NG Stage):

(4) $${\left[-In(1-\alpha )\right]}^{\frac{1}{n}}=K_{1}(t-t_{0})={K_{1}}^{’}(t-t_{0})$$

Phase boundary reaction (I stage)

(5) $$\left[1-{\left(1-\alpha \right)}^{\frac{1}{3}}\right]=K_{2}r^{-1}(t-t_{0})={K_{2}}^{’}(t-t_{0})$$

Diffusion (D stage)

(6) $${\left[1-{\left(1-\alpha \right)}^{\frac{1}{3}}\right]}^{2}=K_{3}r^{-2}(t-t_{0})={K_{3}}^{’}(t-t_{0})$$

where *α* is the degree of hydration, *K*_1_, *K*_2_, and *K*_3_ are the reaction rate constants of the three hydration reaction processes, respectively, *t*_0_ is the end time of the induction period, *n* is the reaction order, and *r* is the particle diameter participating in the reaction.

From the above Kondo equation and the Kstulovic–Dabic model, it can be known that the hydration degree of cement-based materials at various stages must be calculated first when calculating the hydration kinetics of cement-based materials at different stages. Knudson proposed a formula to calculate the kinetics of hydration, which can calculate the degree of hydration, as shown in Equations (7) and (8).

(7) $$\frac{1}{Q}=\frac{1}{Q_{\max}}+\frac{t_{50}}{Q_{\max}(t-t_{0})}$$

(8) $$Q=\frac{Q_{\max}(t-t_{0})}{(t-t_{0})+t_{50}}$$

where *Q* is the cumulative heat release, *Q*_max_ is the theoretical maximum heat release, and *t*_50_ is the time required for the cumulative heat release to reach *Q*_max_/2.

The Knudson equation shows that the ratio of the heat released at different times during the hydration process to the total heat is the degree of hydration, as shown in Equation (9).

(9) $$\alpha =\frac{Q_{1}}{Q_{\max}}$$

**Figure 4:**
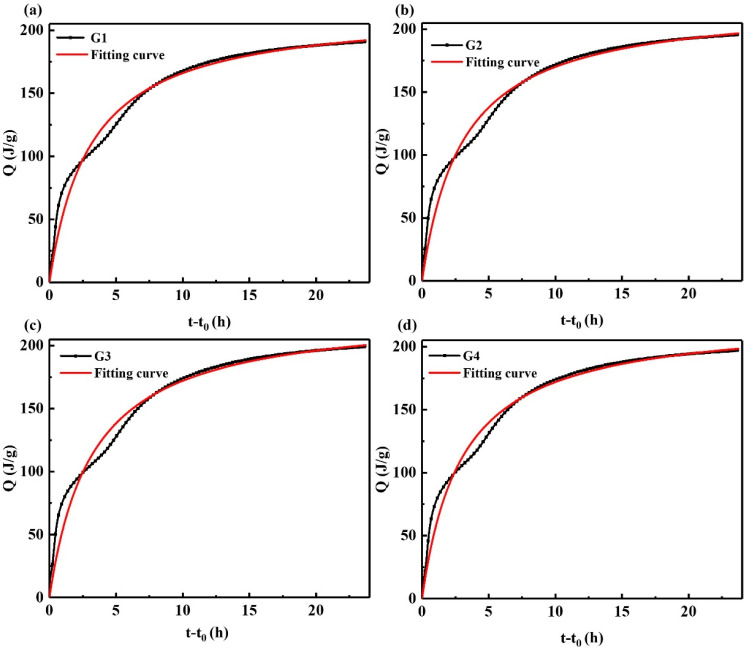
Knudsen equation linear fitting to calculate *Q*_max_; (**a**) G1, (**b**) G2, (**c**) G3, (**d**) G4.

Based on the Knudson equation, the SAC hydration exothermic process was fitted, as shown in Figure 4, and the fitting results are shown in Table 4.

**Figure 5:**
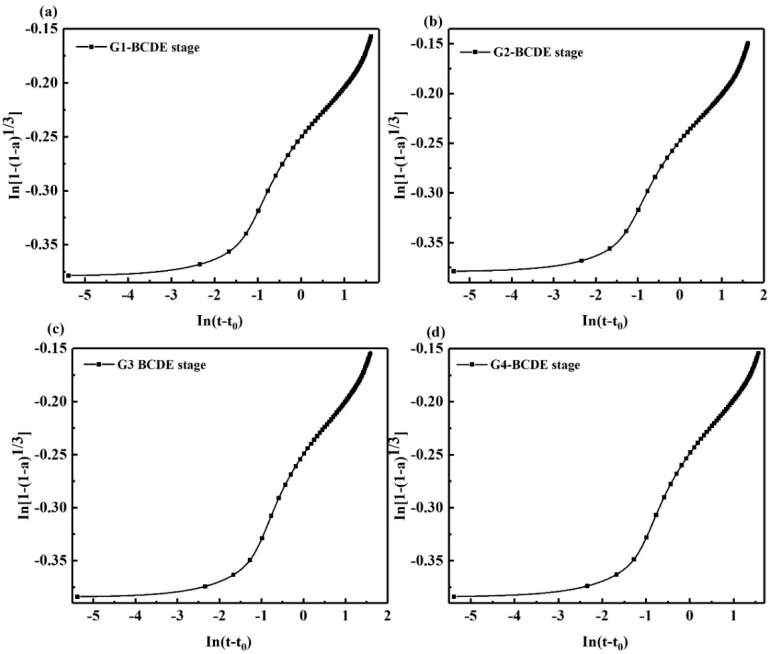
ln [1 − (1 − α)^1/3^] − ln(t − t_0_) curve of SAC in hydration acceleration stage (BCDE stage); (**a**) G1, (**b**) G2, (**c**) G3, (**d**) G4.

It can be seen from Table 4 that the fitting correlation coefficient *R*^2^ of each group is more significant than 0.97, indicating that the relevant effect is excellent, and it can reflect the hydration heat release. When the hydration exotherm is 24 h, the cumulative heat of each group is 189.3 J/g, 195.1 J/g, 199.7 J/g, and 196.5 J/g, respectively. The *Q*_max_ of each group obtained by Knudsen equation fitting were 216.9 J/g, 222.1 J/g, 227.6 J/g, and 223.3 J/g, respectively. According to Equation (9), the hydration degrees of each group were calculated as 87.27%, 87.84%, 87.74%, and 88.00%, respectively, indicating that the addition of GNPs improves the hydration degree of each group of samples. According to Equation (2), the curve of the hydration accelerated stage of the sample is shown in Figure 5. It is challenging to perform linear fitting directly on the curve, and the curve of the hydration accelerated stage contains two stages, and there is a turning point.

**Figure 6:**
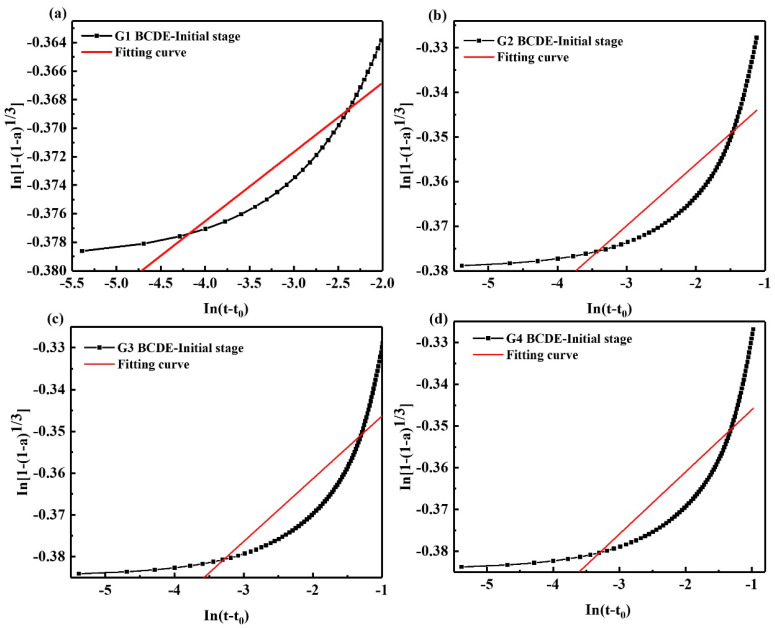
Kondo equation piecewise fitting hydration acceleration initial stage (BCDE-initial stage); (**a**) G1, (**b**) G2, (**c**) G3, (**d**) G4.

As shown in Figure 6 and Table 5, after 1 h of hydration, the hydration entered the acceleration period, and two exothermic peaks appeared successively at 1.339 h and 5.755 h. The hydration acceleration stage of SAC can be divided into two stages, the acceleration initial stage and the acceleration final stage. In the acceleration initial stage, the *N* value is 206.186. The diffusion–reaction mainly controls the hydration process. The clinker dissolves slowly, the Ca^2+^ concentration in the solution increases, and the hydration products such as AFt begin to nucleate and crystallize. At this time, it is in the initial stage of crystal growth, the hydration reaction rate is slow, and the formation resistance of hydration products is enormous. With the progress of the hydration reaction, the number of crystal nuclei increases, the Ca^2+^ in the cement paste increases significantly, many hydration products are formed. At the exothermic peak, with the continuous growth of AFt crystals, the surface of the cement clinker is gradually covered by the coating layer of hydration products, resulting in the slowing down of the clinker dissolution rate and the increase in the formation resistance of hydration products. It can be seen that after the addition of GNPs, the *N* value of each group decreased in the acceleration initial stage, indicating the resistance of the hydration reaction decreased, and the reaction was easy to proceed, suggesting that GNPs promoted the hydration reaction of SAC. In addition, the *K* value increased, indicating the hydration reaction rate is faster, and the reaction occurs easier. After adding GNPs, the *K* value of each group rises, indicating that the hydration reaction rate of each group is accelerated, and GNPs accelerate the hydration reaction of SAC.

**Figure 7:**
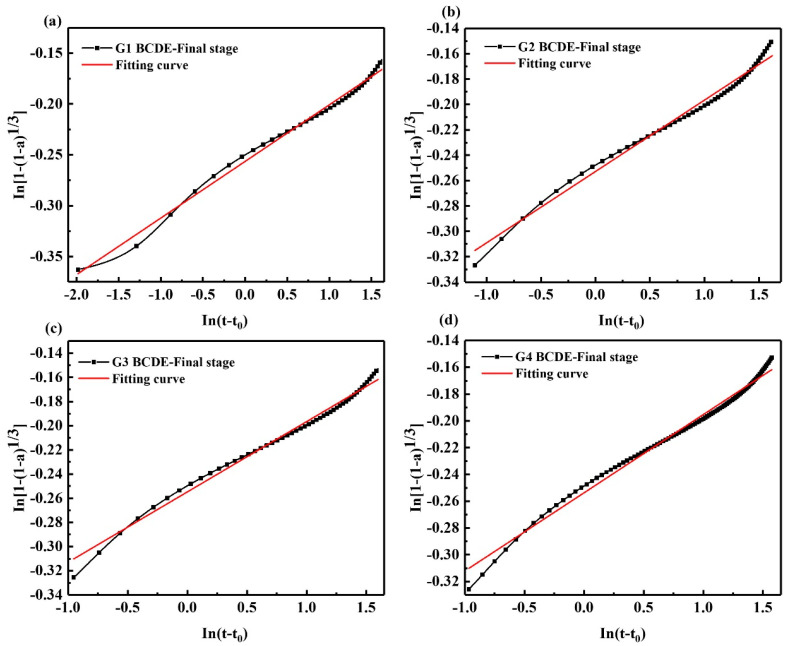
Kondo equation piecewise fitting hydration acceleration final stage (BCDE-final stage); (**a**) G1, (**b**) G2, (**c**) G3, (**d**) G4.

As shown in Figure 7 and Table 6, the coating layer is damaged along with the increased osmotic pressure inside and outside the coating layer, and the hydration enters the acceleration final stage. Compared with the acceleration initial stage, the *N* value decreases to 17.976, the resistance of the hydration reaction becomes smaller, the exposed cement clinker dissolves more Ca^2+^, and hydration products such as AFt have generated again, corresponding to the appearance of the second exothermic peak. However, due to the limited damage to the coating layer, the cement clinker has been dissolved in large quantities, and the amount of Ca^2+^ released is limited. On the other hand, the formed AFt also limits the formation and growth of new AFt. Therefore, the second exothermic peak is smaller than the first exothermic peak. It can be seen that after adding GNPs, the *N* value decreased from 17.976 to 17.114, indicating that the resistance of the hydration reaction became smaller; GNPs promoted the hydration reaction and generated hydration products. The *K* value increased from 0.0099579 to 0.0130089, indicating that the hydration reaction rate increased, and GNPs accelerated the hydration exothermic rate of SAC.

**Figure 8:**
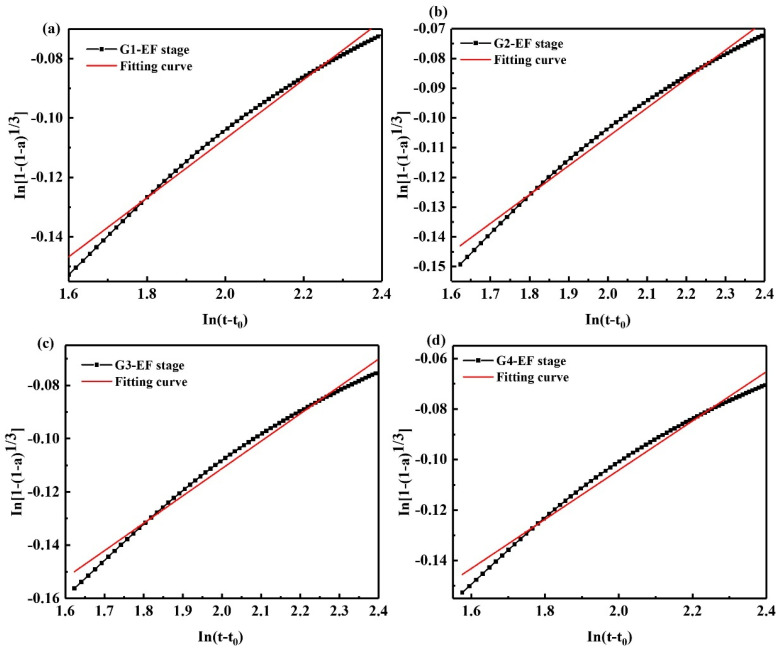
Kondo equation piecewise fitting hydration deceleration stage (EF stage); (**a**) G1, (**b**) G2, (**c**) G3, (**d**) G4.

As shown in Figure 8 and Table 7, when the hydration enters the deceleration stage, compared with the acceleration stage, the *N* value decreases and the *K* value increases, which indicates that the hydration reaction rate is faster, and the hydration reaction resistance is small. However, after the acceleration period, the clinker is consumed in large quantities, and the formation of AFt has become stable. This indicates that another hydration reaction quickly occurred in the deceleration period, and the rate is faster. According to the literature, in the deceleration period of SAC hydration, the hydration product wraps the clinker, the ions are difficult to dissolve, the SO_4_^2−^ is reduced, and part of the AFt would transform and form AFm [37,38,39].

**Figure 9:**
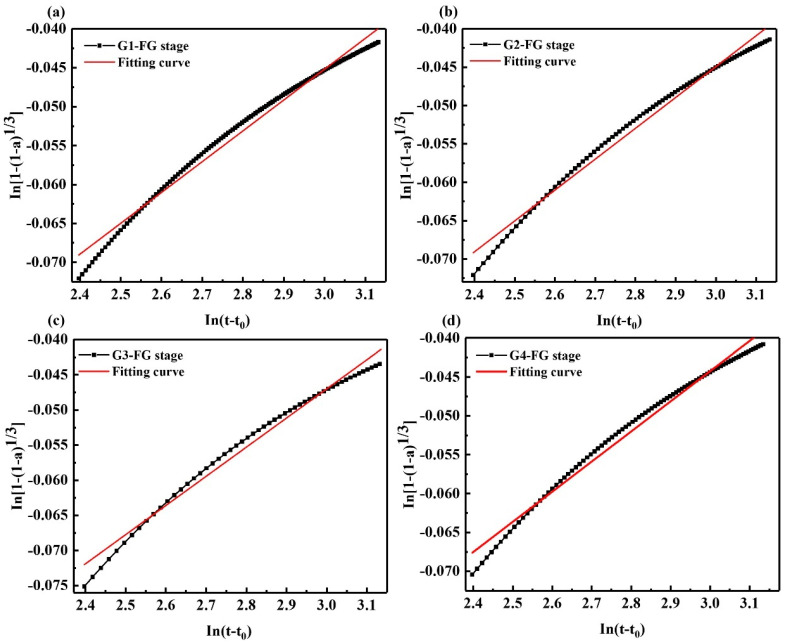
Kondo equation piecewise fitting hydration stable stage (FG stage); (**a**) G1, (**b**) G2, (**c**) G3, (**d**) G4.

As shown in Figure 9 and Table 8, after the hydration enters the stable stage, compared with the deceleration stage, the *N* value increases and the *K* value decreases, indicating that the hydration reaction resistance is large, and the hydration reaction rate is slow. The diffusion–reaction mainly controls this stage, and the paste porosity is further reduced. The structure is more compact, water molecules can hardly pass through the hydration product, and the paste almost loses its plasticity.

## 4. Conclusions

This paper studied the effect of GNPs on the hydration of sulfoaluminate cement (SAC), and the hydration kinetic model of SAC was established. The hydration kinetic equation of sulfoaluminate cement is $y=\frac{216.9x}{3.07+x}$, the hydration exotherm of sulfoaluminate cement is mainly concentrated in the first 24 h; when the hydration exotherm is 24 h, the cumulative heat of each group is 189.3 J/g, 195.1 J/g, 199.7 J/g, and 196.5 J/g, respectively. The *Q*_max_ of each group, obtained by Knudsen equation fitting, was 216.9 J/g, 222.1 J/g, 227.6 J/g, and 223.3 J/g, respectively. The hydration degrees of each group were calculated as 87.27%, 87.84%, 87.74%, and 88.00%, respectively; the exothermic peaks are advanced, indicating that the addition of GNPs improves the hydration degree of each group of samples, generating more hydration products. The hydration process of SAC mainly includes five stages: the initial stage, induction stage, acceleration stage, deceleration stage, and stable stage. Different from the hydration reaction of Portland cement, the hydration reaction of SAC is mainly a diffusion–reaction process. In addition, according to the changes in hydration kinetic parameters, it is speculated that during the hydration deceleration period, the reduction in sulfate ions will lead to the transformation of AFt. In the following experiment, more test data verification is required, including changes in the content of hydration products, changes in ion concentration in the solution, and changes in pH value.

## Figures and Tables

**Table 1:** Physical property of SAC.

Loss on Ignition/%	Setting Time/min		Specific Surface Area (m^2^/kg)	Density (g/cm^3^)	Flexural Strength/MPa		Compressive Strength/MPa	
	Initial Setting	Final Setting			3d	28d	3d	28d
1.4	32	56	≥350	3.1	6.3	7.2	42.5	47.2

**Table 2:** Chemical compositions of SAC (wt%).

Type	Fe_2_O_3_	Al_2_O_3_	TiO_2_	SO_3_	MgO	SiO_2_	CaO
SAC	2.15	16.34	1.31	12.45	2.52	19.15	46.08

**Table 3:** Characteristics of GNPs.

Type	Thickness/nm	Surface Area/m^2^·g^−1^	Density/(g/cm^3^)	Particle Diameters/μm	Purity/%
xGnP-M25	6–8	120–150	<3	25	>99.5

**Table 4:** Hydration kinetic parameters of SAC.

Codes	Fitted Equation	*Q*_max_ (J/g)	*t*_50_ (h)	*R* ^2^
G1	$y=\frac{216.9x}{3.07+x}$	216.9	3.07	0.98053
G2	$y=\frac{221.1x}{3.06+x}$	222.1	3.06	0.97725
G3	$y=\frac{227.6x}{3.22+x}$	227.6	3.22	0.97504
G4	$y=\frac{223.3x}{2.99+x}$	223.3	2.99	0.98132

**Table 5:** Results of Kondo equation piecewise fitting hydration acceleration initial stage.

Codes	Fitted Equation	*N*	*K*	*R* ^2^
G1	y=−0.35713+0.00485x	206.186	-	0.84435
G2	y=−0.32861+0.01376x	72.674	-	0.72604
G3	y=−0.3313+0.01502x	66.578	-	0.71423
G4	y=−0.33116+0.01492x	67.024	-	0.71568

**Table 6:** Results of Kondo equation piecewise fitting hydration acceleration final stage.

Codes	Fitted Equation	*N*	*K*	*R* ^2^
G1	y=−0.25642+0.05563x	17.976	0.0099579	0.99026
G2	y=−0.25278+0.05621x	17.790	0.0111416	0.98593
G3	y=−0.25472+0.05814x	17.199	0.0125109	0.98976
G4	y=−0.25371+0.05843x	17.114	0.0130089	0.98982

**Table 7:** Results of Kondo equation piecewise fitting hydration deceleration stage.

Codes	Fitted Equation	*N*	*K*	*R* ^2^
G1	y=−0.30578+0.0994x	10.060	0.0461315	0.98762
G2	y=−0.30068+0.09716x	10.292	0.0452890	0.98736
G3	y=−0.31688+0.10283x	9.725	0.0458862	0.98699
G4	y=−0.29897+0.09741x	10.266	0.0464587	0.98487

**Table 8:** Results of Kondo equation piecewise fitting hydration stable stage.

Codes	Fitted Equation	*N*	*K*	*R* ^2^
G1	y=−0.16392+0.03958x	25.265	0.0158920	0.98473
G2	y=−0.16553+0.0402x	24.876	0.0162825	0.98484
G3	y=−0.17143+0.04148x	24.108	0.0160373	0.98409
G4	y=−0.1605+0.03875x	25.806	0.0158992	0.98510
